# Effect of Mechanically Activated Nepheline-Syenite Additive on the Physical–Mechanical Properties and Frost Resistance of Ceramic Materials Composed of Illite Clay and Mineral Wool Waste

**DOI:** 10.3390/ma16144943

**Published:** 2023-07-11

**Authors:** Jolanta Pranckevičienė, Ina Pundienė

**Affiliations:** Laboratory of Concrete Technology, Institute of Building Materials, Vilnius Gediminas Technical University, Linkmenų Str. 28, 08217 Vilnius, Lithuania; jolanta.pranckeviciene@vilniustech.lt

**Keywords:** ceramics, structural parameters, nepheline-syenite, mineral wool melt waste, frost resistance

## Abstract

This study investigates the coupling effect of mechanically activated nepheline-syenite (NS) and mineral wool melt waste (MWMW) on the physical–mechanical properties of a ceramic body. The results indicate that an optimal amount (10–20%) of NS additive promotes the formation of the smallest pore size from 0.001 to 0.01 µm, as well as improves physical, mechanical, and durability properties of the ceramic samples with MWMW, when fired at temperatures between 1000 and 1080 °C. As the NS content increases, the composition becomes more alkaline, leading to enhanced vitrification and the formation of a glass phase during firing. This reduces open porosity, modifies pore size distribution, and enhances compressive strength and frost resistance. An NS content of 15% produces the best results, increasing the smallest pore fraction and yielding favourable properties, such as reduced open porosity, water absorption and density, increased compressive strength, and does not affect the linear shrinkage. The frost resistance test demonstrates that the coupling effect of NS additive and MWMW improves the samples’ resistance to freeze–thaw cycles, with the best performance observed at 15% NS content. The study also highlights the usefulness of structural parameters and ultrasound testing for assessing and predicting the frost resistance of ceramic samples.

## 1. Introduction

The production of ceramic building materials is distinguished by waste-free technologies, vast possibilities of using various wastes, and associated technogenic (artificial) products from other industries [1]. It can be said that this is the only industry capable of using numerous and large-tonnage wastes from many other industries in the ceramic production process [2]. However, the waste utilisation rate remains low. Moreover, modification of technology and careful selection of compositions can make it possible to obtain high-quality ceramics with controlled porosity that meet modern requirements for performance properties. A review of the literature showed that additives of various wastes—cullet [3], scrap glass [4], volcanic rocks, and some industrial wastes, such as cupola slag, metallurgical slag, various ashes, catalyst waste from the catalytic cracking of petroleum products, waste from the production of mineral wool, etc., improve the characteristics of ceramic products [5,6,7,8]. In the research [9,10,11], the use of waste from the production of mineral wool as a substitute for sand in the composition of ceramics has been proposed. This is due to the fact that mineral wool waste melts at temperatures above 1100 °C. The use of mineral wool can lead to an increase in the compressive strength of ceramic samples. However, it significantly increases the water absorption of the samples, which could negatively affect their functional properties, particularly their frost resistance [10,12].

Today, there is a need to obtain durable porous materials that provide satisfactory frost resistance. The existing methods for increasing a ceramic body’s frost resistance have several disadvantages [13]. The introduction of burnout additives into the composition does not, in all cases, increase the frost resistance, and at the same time, the appearance of the products deteriorates due to the formation of dark spots [14,15,16,17]. An increase in the firing temperature, but not for all types of raw clay materials, yields a positive effect and is disadvantageous from an ecological and economic standpoint. For example, in the patent [18], the frost resistance of bricks is increased by introducing up to 40% of mechanically activated fly ash into the batch. The effect was achieved due to fine grinding of a part of the ash to obtain pores with a diameter of 10–100 microns and open micropores with a diameter of less than 1 micron. The frost resistance is determined by the pore size, pore type, and pore distribution, which depend on the firing temperature [19]. The presence of small pores, where water can intrude only during boiling, may improve frost resistance, although this assumption is controversial and it has been put in doubt by recent studies [20]. According to [21], the frost resistance is influenced not only by pore volume, but also by the median pore radius. The highest frost resistance is reached when the pore volume decreases, and at the same time, the pore radius median increases.

Frost resistance is primarily determined by the material’s pore space structure [13]. The freezing of water in the pores of the ceramic body leads to the appearance of tensile stresses inside the material. Ensuring frost resistance is achieved not only by a given porosity, but also by the strength of the pore walls [22,23,24,25,26,27]. According to authors [28,29], the pores, according to the diameter, are conventionally divided into safe ones (less than 0.1 microns, water does not enter them; therefore, it does not freeze in the winter period of operation); hazardous (from 0.1 to 200 microns, where water freezes at low temperatures); and reserve (more than 200 microns, in which water is not retained). Other authors [30,31] prove that small pores (<0.2 µm) have little effect on the frost resistance of brick, while the main contribution brings medium size pores (>1 µm) [32]. The research offered an even more detailed pore size distribution [21]. The authors have divided pores into five groups (<0.1 µm, 0.1–1 µm, 1–3 µm, 3–10 µm, and >10 µm). They concluded that pores with a diameter <0.1 µm do not affect the resistance of brick to freeze–thaw cycles. Pores with diameters in the 3–10 µm range possess the main influence.

Most pores within a ceramic body are interconnected by capillaries through which water can move. Pores smaller than 0.1 microns retain air and do not fill with water. Therefore, the meticulous selection of compositions and components and the granulometry obtained through mechanical activation of these components can enhance ceramics’ pore structure and frost resistance. In some instances, the frost resistance of ceramic materials can be improved by introducing a fluxing additive into the composition of the masses, which contains a significant amount of oxides of alkaline earth metals. Natural alkaline aluminosilicates, such as nepheline-syenite, can be used for the regulation of the pore structure of fluxing additive [33,34,35,36,37,38].

Nepheline is a feldspathoid mineral of composition (Na, K) AlSiO_4_ and usually forms small grains, which are inter-crystallised with the feldspar [39,40,41]. Nepheline-syenite is a silica-undersaturated igneous rock containing feldspars and feldspathoids (nepheline, leucite, etc.), which is similar in its medium- to coarse-grained appearance to granite, with deficiency in silica. In addition, a high proportion of the alkalis, sodium, and potassium, as well as a high proportion of alumina and its properties help in achieving a comparable fluxing action [35]. The technological role of fluxes in ceramic bodies mainly consists of forming a liquid phase during firing by melting alkali minerals. This liquid phase at high temperatures penetrates the porous structure of unfired ceramic bodies and densifies the structure [42]. As pointed out in [40], very high fusibility is exhibited by quartz-free and feldspathoid-bearing fluxes that exhibit very high fusibility, which are remarkably rich in feldspars, such as nepheline-syenites and phonolites.

A characteristic alumina/fluxes weight ratio in feldspars averages 1.4; in nepheline-syenites and phonolites, this ratio is about 1.1 and 0.5 in rock-related fluxing wastes [33,34,43,44,45]. It is known that the alumina/fluxes ratio affects the viscosity of the liquid phase formed during firing [46].

Nepheline-syenite can be introduced as a fluxing additive in the production of ceramics and glass [35,47,48]. The addition of nepheline-syenite increases the amount of the liquid phase during the firing process of a ceramic body and decreases its viscosity [37,44,49]. It also alters the ceramic body’s porosity by partially filling the open pores [50]. Research [51] has demonstrated that the sintering of ceramic materials from low-plastic industrial waste and ground nepheline-syenite additive results in closed pores ranging from about 0.04 to 4.4 mm, creating numerous closed macropores within the ceramic body. Despite the low firing temperature (1050–1150 °C) used in porous or red-firing bodies (for example, illite clay bodies), fluxing additives are still used in these ceramics. In these instances, fluxing additives serve a dual role—acting as very fine fillers and fluxing agents [52].

The addition of ground nepheline-syenite influences the porosity of the ceramic body by modifying the pore size [37,53]. These changes in pore size within the ceramic body [10,34] improve the physical and mechanical properties of the ceramic body. However, more detailed studies on how nepheline-syenite changes the structure of the ceramic body as well as the distribution of pores by size, and thereby manages the physical and mechanical properties have not been sufficiently carried out. In addition, the effect of mechanically activated nepheline-syenite, especially when combined with waste from mineral wool production can provide new properties to the ceramic body, improving pore structure, mechanical properties, and its consequent frost resistance. As a result, extensive research has been undertaken to understand the influence of mechanically activated nepheline-syenite additive on the ceramic body, consisting of low-melting illite clay and mineral wool production waste on the physical–mechanical properties in order to enrich the application. The study encompasses an examination of the pore structure and frost resistance of the ceramic body, enabled through various methods. It involves calculated structural parameters, the Maage factor, and a frost resistance test, which are augmented with measurements of compressive strength and ultrasonic pulse velocity (UPV), as well as damage indicator calculations. These methods collectively assist in the development of testing procedures for evaluating the frost resistance of the ceramic body.

## 2. Materials and Methods

This study used the mineral wool melt waste (MWMW) and the fluxing additive mechanically activated nepheline-syenite (NS) to investigate the low-melting illite clay obtained from the factory of ceramic products. The clay mineral composition presents illite, chlorite, calcite, silica, dolomite, and feldspar minerals, as pointed out in previous research [10]. Before using the clay, it was dried at 100 (±5 °C), and then ground in a laboratory ball mill. The granulometric and chemical composition of components are presented in Table 1 and Table 2.

**Figure 1:**
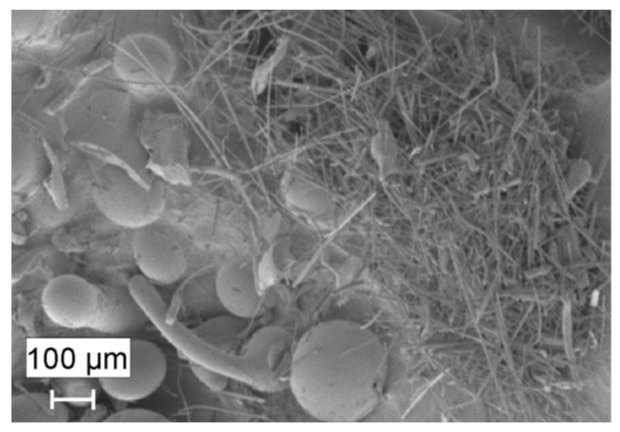
The SEM image of the MWMW.

MWMW consists of mineral wool fibres, pieces of melt, beads, and a small amount of organic binder material (1–2%) (Figure 1), which are neutralised according to the methodology [11] before using MWMW. Therefore, MWMW can be used instead of natural sand. The average particle size of the MWMW is 62 μm and the specific surface area is 2050 cm^2^/g.

NS, also known as syenite alkali aluminium concentrate, is produced by “Apatit” JSC by reprocessing apatite-nepheline ore. This process results in a product characterised by a high concentration of alkali metals, making it a useful additive in various industrial applications. NS used in this study was ground in a ball mill for 4 h. The milling resulted in an average particle size of 9.79 μm and a specific surface area of 8100 cm^2^/g, effectively enhancing its reactivity and potential interaction with other components in the ceramic body.

**Figure 2:**
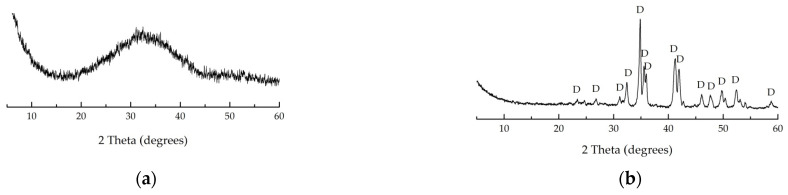
XRD of the MWMW. (**a**) Non-fired; (**b**) after firing at 1000 °C. D—diopside (CaMgSi_2_O_6_).

**Figure 3:**
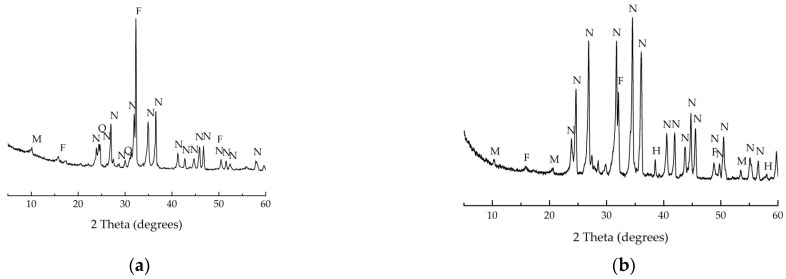
XRD of the NF. (**a**) Non-fired; (**b**) after firing at 1000 °C. F—feldspars (NaAlSi_3_O_8_); M—muscovite (KAl_2_[(OH, F)_2_AlSi_3_O_10_]); N—nepheline (KNa_3_[AlSiO_4_]_4_); Q—quartz (SiO_2_).

Figure 2 and Figure 3 show X-ray diffraction patterns of non-treated MWMW and NS fired at 1000 °C MWMW and NS. Diopside predominates in the mineralogical composition of MWMW, and nepheline, quartz, muscovite, and feldspar in the mineralogical composition of NS.

The compositions of the forming masses and chemical composition of the forming masses SiO_2_/Al_2_O_3_ and Na_2_O + K_2_O/SiO_2_ ratios in the forming masses are presented in Table 3 and Table 4. Additionally, SiO_2_/Na_2_O + K_2_O and Al_2_O_3_/Na_2_O + K_2_O ratios, that assist in following the pointed oxides ratio changes in compositions, are presented in Table 4. We can see that the increase in NS in composition SiO_2_/Na_2_O + K_2_O ratio decreases by 2.74 times, and Al_2_O_3_/Na_2_O + K_2_O ratio by 2.36 times.

The reference forming mass composition C_W_, consisting of dry clay and MWMW, and five compositions (Table 3), containing NS in amounts of 5%, 10%, 15%, 20%, and 25% by weight, named C_W_N_5_, C_W_N_10_, C_W_N_15_, C_W_N_20_, and C_W_N_25_ were prepared. The dry components were initially mixed in a special planetary mixer with a capacity of 20 L for 10 min. After mixing, moulding masses were wetted (i.e., 20–22%) until a consistency suitable for the moulding mixture was obtained. The prepared mass was conditioned for 3 days at 95 ± 5% to achieve uniform humidity distribution in the forming mass. After 3 days, 50 × 50 × 50 mm specimens were formed. Fifty specimens were prepared for each forming mixture, and for each firing temperature, six specimens were used. The specimens were maintained for 3 weeks under natural laboratory conditions. Thereafter, they were dried to the constant mass at 105 ± 5 °C temperature in this regime: Dried in an oven at a temperature of (60 ± 5) °C for one day and at a temperature of (105 ± 5) °C for a second day. Dried specimens were fired at 1000, 1020, 1040, 1060, and 1080 °C temperatures. The overall firing duration is 36 h, while maintaining the highest firing temperature at 8 h.

The specific surface of the material used was determined by Blaine’s method. The physical and mechanical properties of ceramic specimens were tested according to the following standardised testing methods: EN 772-13 [54] for density testing; EN 772-21 [55] for water absorption testing; EN 772-1 [56] for compressive strength testing. Cilas 1090 L analyser was used to analyse the particle size distribution in raw materials. Dilatometry tests were carried out by dilatometer Linseis L76, with a controlled heating rate of 4 °C/min. A Linseis STA PT-1600 derivatograph was used for differential thermographic analysis at 10 ± 1 °C/min.

The development of the structure of the sample after firing and structure changes in the samples during freeze–thaw cycles was evaluated using the ultrasonic pulse velocity (UPV) method by the tester Pundit 7. Tested samples were placed between two ultrasonic transducers (transmitter and receiver) operating at 54 kHz. The transducers were pressed against the specimens at two directly opposite points. Vaseline was used to ensure good contact. The ultrasonic pulse velocity (V) was calculated using Equation (1):

V = l/τ × 10^6^ (1)

where l is the distance between the cylindrical heads and τ is the time of pulse spread.

Porosimeter Pore Master PM33-12 tested the distribution of pores in the ceramic body, and the testing sample was obtained from the internal layer of the specimen. The microstructure was tested by the scanning electron microscope SEM JSM-7600F (JEOL, Tokyo, Japan).

The forecast of frost resistance according to several physical parameters and calculated structural parameters of ceramic products is appropriate and confirmed in several studies [57,58,59,60,61]. The study employed three different methods to predict the frost resistance of ceramic bodies indirectly. The first one is the method developed by Maage [57,62], in which the potential frost resistance is evaluated with a single numerical quantifier determined according to the following formula:

Fc = 3.2/PV + 2.4 × P_3_ (2)

where Fc is the frost resistance number; PV is the pore volume in cm^3^/g; P_3_ is the percentage of the pores with diameters larger than 3 µm.

Another method is based on the calculation of structural parameters—effective porosity We, total open porosity, reserve pore volume P_R_, relative wall thickness D of pores, and capillaries of ceramic bodies. These structural parameters were determined according to the methodology in [59,61].

Process analysis can help in better characterising the pore structure of samples and predicting the frost resistance of ceramic products. The effective porosity of a ceramic body determines the amount of actively working pores and capillaries. Total open porosity defines a ceramic body’s overall open porous space in both macrostructure and microstructure dimensions. The pore volume reserve defines the number of reserve pores and capillaries, which are difficult for water or plastic ice to penetrate. The larger the pore volume reserve, the greater the exploitation of frost resistance of ceramics. The relative wall thickness of pores and capillaries defines the suitable thickness of the wall of pores and capillaries. The greater the thickness of the wall of pores and capillaries, the greater the frost resistance of ceramic products.

The third technique involves using ultrasonic pulse travel times to monitor frost damage in the sample. Prior to testing, the state of each sample is assessed, and after being subjected to 100, 200, and 300 frost–thaw cycles, the samples are re-evaluated. The time required for the pulse to pass through the sample is measured along the longitudinal direction at the exact sensor locations each time. The pulse travel times through the sample are then linked to Young’s modulus of the materials. Often, the relative dynamic modulus is transformed into the damage indicator Ω as a way to assess the level of damage incurred by the frost [63,64]:

Ω = 1 − E/E_0_ = 1 − (t_0_/t)^2^ (3)

where Ω is the damage indicator; E, E_0_ (Pa) and t, t_0_ (s) are, respectively, Young’s modulus and the pulse travel time after and before frost–thaw cycles. The evaluation based on Young’s modulus is adapted from the standard for natural stone [65]. However, since all three employed methods allow for the evaluation of qualitative parameters, and not a quantitative assessment, for an additional evaluation of the resistance of the sample to freeze–thaw cycles, the changes in compressive strength (expressed as the ratio of compressive strength of the samples before and after freezing) were studied.

A direct test of frost resistance of ceramic samples was conducted according to the CEN/TS 722-22 by a one-sided freeze–thaw method. One part of the samples (compositions C_W_, C_W_N_5_, C_W_N_15_, and C_W_N_25_) was tested for 100 frost–thaw cycles, the second for 200 frost–thaw cycles, and the third for 300 frost–thaw cycles.

## 3. Results

### 3.1. Dilatometric Analysis

**Figure 4:**
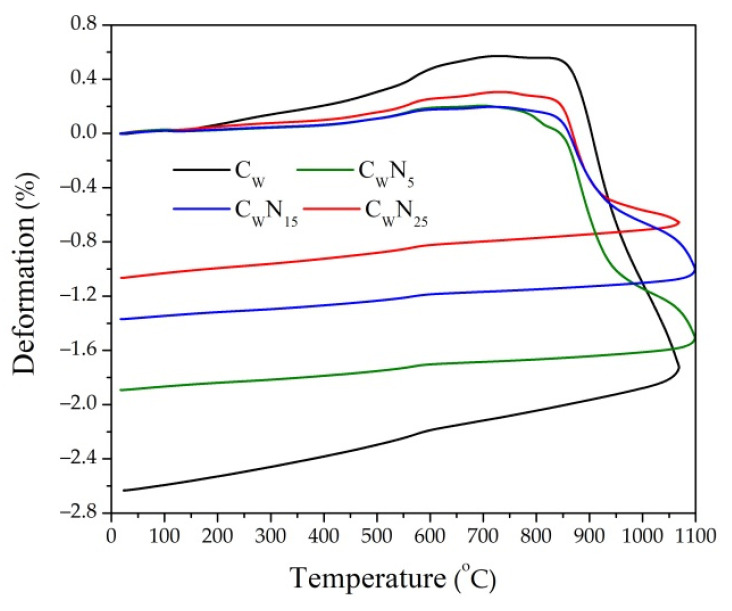
Dilatometric curves of samples: C_W_—sample without NS, C_W_N_5_—sample containing 5% of NS, C_W_N_15_—sample containing 15% of NS, C_W_N_25_—sample containing 25% of NS.

**Figure 5:**
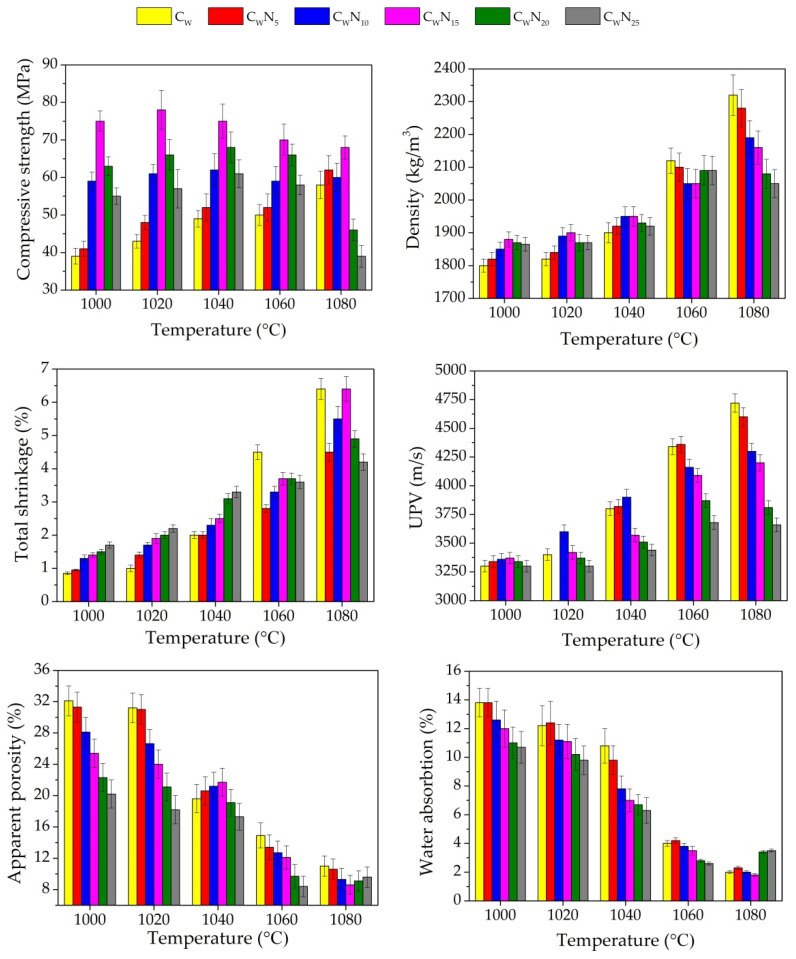
Basic physical and mechanical properties of ceramic samples.

The dilatometric analysis of four compositions (reference and with 5, 15, and 25% of NS amount), reflecting sintering kinetics of the samples, was conducted (Figure 4). Dilatometric curves of all compositions exhibit a slight continuous expansion during firing until 500–550 °C temperature. However, the expansion of samples with an increase in NS amount gradually decreases, which can be related to the reduction in the SiO_2_/Na_2_O + K_2_O ratio in the compositions with NS from 13.76 to 6.73. Starting at 530–550 °C temperature, an expansion of ceramic samples increases until approximately 600 °C temperature, mainly due to the allotropic transformation of α-quartz into β-quartz [34]. In addition to the quartz transformation process, dehydroxylation of the clay mineral illite [66] presented in the tested clay, occurs between 500 and 700 °C temperature in all compositions. In temperatures above 600 °C, expansion gradually loses intensity until it reaches 770–860 °C temperature, which is the moment when these materials reach their maximum expansion and new crystal and glass phase formation starts, decreasing the open porosity as the liquid phase fills the pores. For the C_W_ sample with SiO_2_/Na_2_O + K_2_O ratio of 19.61, the expansion process finished at 860 °C temperature; for samples with NS and a correspondingly lower SiO_2_/Na_2_O + K_2_O ratio, the expansion process finished at a lower temperature: For C_W_N_5_ at 850 °C, for C_W_N_15_ at 820 °C, for C_W_N_25_ at 780 °C. According to the previous studies [67] describing thermal evolutions of clay minerals under thermal dilatometric measurement, dehydroxylated illite presents a lath-like morphology with the appearance of elongated spinel in Al-rich or Mg-rich micro-domains (an octahedral relict after the destruction of the illite structure). Liquid phases also occur in the Si-rich micro-domain (the tetrahedral relict and the K+ from illite interlayer). As pointed out in [37], alkali metals and silica increase the viscosity, resulting in lower expansion. The maximum expansion of reference composition C_W_ is (0.59%), and for compositions with NS, expansion is noticeably lesser and varies from 0.30 to 0.20%. This decrease in expansion deformations may be caused by a high amount of Na_2_O and K_2_O, reaching 19.73% in NS composition. According to [34], smaller amounts of alkali metals (i.e., up to 15%)) cause no significant decrease in the expansion deformation caused. This indicates that the NS additive, rich in alkali metals, acts as a fluxing additive and reduces the expansion deformations by 65.5% compared to the reference sample. Therefore, the dilatometric analysis showed that the NS affected the expansion and shrinkage deformations of the ceramic body and could result in a smaller amount of open pores and the formation of micro-cracks. The linear shrinkage deformation of the reference C_W_ sample at 1080 °C reached 2.63%, whereas the linear shrinkage of samples with NS decreased with an increase in NS amount in the composition: for C_W_N_5_—1.89%, for C_W_N_15_—1.37%, and C_W_N_25_—1.07%. These studies are confirmed by the absorption, ultrasonic pulse velocity, and porosity research presented in (Figure 5).

### 3.2. Physical and Mechanical Properties

The results obtained for the density, compressive strength, linear shrinkage, apparent porosity, water absorption, and ultrasonic pulse velocity of the samples fired at 1000–1080 °C are shown in Figure 5. By analysing the data, it is evident that the density of reference C_W_ sample is the lowest after firing at 1000–1040 °C. The addition of NS (from 5 to 15%) increases the density of the samples to 4.4% after firing at a temperature of 1000 °C and 1020 °C and by 2.6% after firing at a temperature of 1040 °C. Adding 20–25% of NS noticeably decreases the density of samples, respectively, by 3.6% and 2.6–1.5% at pointed temperatures. The literature shows that the more noticeable fluxing action of fluxing additive occurs at higher firing temperatures [37]. The linear shrinkage with an increase in NS additive amount in composition increases up to 2.2 times after firing at a temperature of 1000 °C and 1020 °C and up to 1.65 times after firing at 1040 °C. The ultrasonic pulse velocity (UPV) research reflects changes in sample density after firing at a temperature of 1000–1040 °C when the amount of NS additive is 5 and 10% in the composition. When the amount of NS increases to 15–25%, UPV in composition decreases, probably due to lower shrinkage deformation in these samples. Apparent porosity and water absorption measurements after firing at a temperature of 1000 °C and 1020 °C show a consistent decrease in these parameters with an increase in the amount of NS in composition. After firing at a temperature of 1040 °C, some increase in apparent porosity (about 1%) is observed for C_W_N_5_–C_W_N_15_ samples. Water absorption consistently decreases with the increase in the composition’s NS amount. Adding NS in the amount of 10–25% significantly increases the proportion of Na_2_O + K_2_O in the composition and, due to the fluxing action, reduces the water absorption of the samples. As it is pointed out in [37,43,46,48], the formation of the vitreous phase by the progressive melting of a fluxing agent is a fast phenomenon, starting from 990 °C in the feldsparoid-based flux, which is mainly accomplished before viscous flow begins densification. The eutectic temperature for potash feldspar with silica starts at around 990 °C and that of soda feldspar at 1050 °C. The lower liquid formation temperature in the potash feldspar is beneficial for reducing the ceramic body firing temperature [37,68].

**Figure 6:**
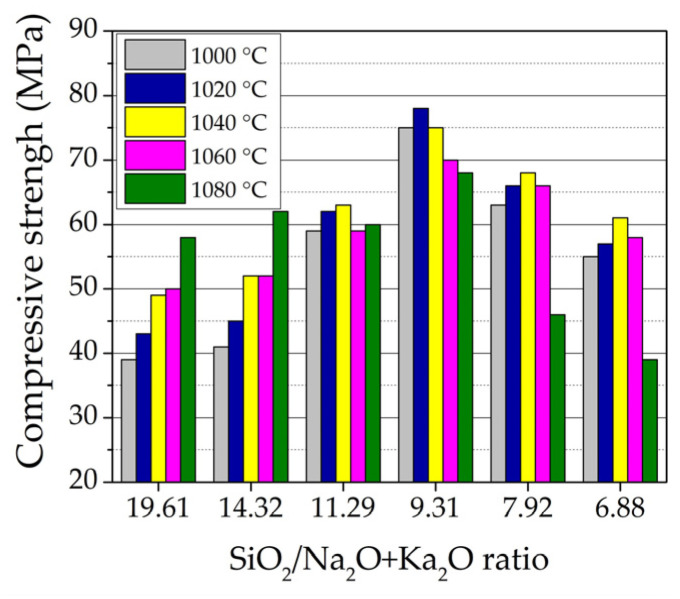
The influence of SiO_2_/Na_2_O + K_2_O ratio in composition on the compressive strength of samples.

The compressive strength of the samples highly depends on the SiO_2_/Na_2_O + K_2_O ratio in the sample composition (Figure 6). With an increased amount of NS to 15% and SiO_2_/Na_2_O + K_2_O ratio decrease from 14.32 to 9.31, respectively, the compressive strength of the samples C_W_N_5_–C_W_N_15_ increased to 5.10–92.3% after firing at a temperature of 1000 °C, and by 4.70–81.0% after firing at a temperature of 1020 °C, and further by 6.1–53.0% after firing at a temperature of 1040 °C in comparison to the compressive strength of the reference sample. With the increasing amount of NS to 20 and 25%, when the SiO_2_/Na_2_O + K_2_O ratio decreases to 7.92 and 6.88, the compressive strength after firing at a temperature of 1000 °C increases by 61.5 and 41.0%, after firing at a temperature of 1020 °C by 53.5 and 32.6%, and after firing at a temperature of 1040 °C by 38.8 and 24.5% compared to the compressive strength of the reference C_W_ sample. The development of mechanical properties in the samples is confirmed by previous studies [10], where it was concluded that NS additive affected the crystallisation of the minerals in the ceramic body, since during firing, NS melted and active K^+^ and Na^+^ ions diffused into the ceramic body. During the diffusion of Na^+^ ions into the ceramic body, the mineral labradorite is formed, and in the case of K^+^ ions, the mineral leucite is crystallised. As reported in [69,70], the formation of leucite minerals in the ceramic body improves the mechanical properties of the ceramic body. It can be concluded that the mechanical strength starts to decrease when the NS dosage is >15%. Considering that this is an unusual phenomenon, a possible reason is the excessive liquid formed, which could yield cracks or uneven shrinkages in the ceramic body [71].

With an increase in firing temperature, the formation of new crystal and liquid phases causes new strong bonds to form between atoms, which increase the samples’ density, compressive strength, and shrinkage. At the same time, open porosity decreases as the liquid phase fills the pores. After firing at 1060 °C, the density of reference C_W_ sample is the highest, reaching 2120 kg/m^3^; the linear shrinkage reaches 4.5%. With the increase in NS (5–15%) in composition, the densities and linear shrinkages reach only 2100–2050 kg/m^3^ and 2.8–3.7% values. For samples with higher NS amounts, the densities increase until 2090 kg/m^3^ and linear shrinkages of the samples until 3.8–3.7%. With an increase in firing temperature, the UPV in the samples increases, reflecting structure densification, but with an increase in NS amount, the UPV in the samples tends to decrease. The same tendencies are observed for apparent porosity and water absorption—with an increase in NS amount in composition and apparent porosity, and decrease in water absorption. The compressive strength of the samples containing 15 and 20% of NS and SiO_2_/Na_2_O + K_2_O ratios of 9.31 and 7.92, respectively, reaches the highest values—70.0 and 68.0 MPa, as pointed in [10], but the further increase in NS until 25% in composition decreases SiO_2_/Na_2_O + K_2_O ratio until 6.88 and the compressive strength of samples until 63.0 MPa.

**Figure 7:**
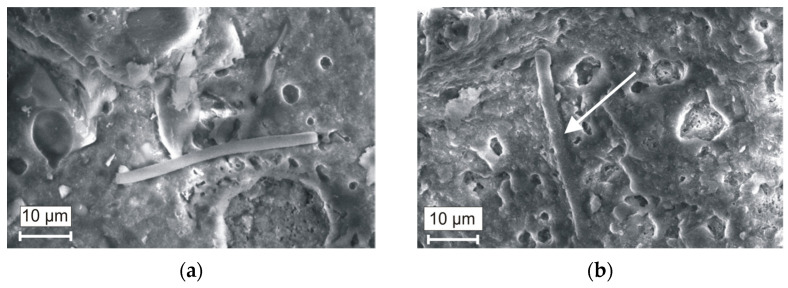
The microstructure of the ceramic bodies after firing at 1060 °C: (**a**) Reference ceramic body and (**b**) ceramic body C_W_N_15_.

By observing the microstructure of the reference sample (Figure 7a) after firing at 1060 °C, it can be seen that MWMW fibre did not melt and the fibre does not firmly adhere to the ceramic matrix. The microstructure of the C_W_N_15_ sample (Figure 7b) significantly differs and is more homogenous with a higher amount of pores. The MWMW fibres are covered with a layer of the liquid phase; in this way, the matrix is reinforced—this reinforcement results in a higher compressive strength of the ceramic body.

After firing at 1080 °C, the densities of reference C_W_ samples and samples containing 5, 10, and 15% of NS further increase. The density of reference C_W_ samples remains the highest—2320 kg/m^3^. The densities of the samples containing 5, 10, and 15% of NS increased until 2290–2150 kg/m^3^. These trends are confirmed by other researchers [38,47,72]. However, for samples with 20 and 25% of NS, a sudden drop in density to 2080 and 2050 kg/m^3^ was observed. Probable swelling of the sample at this temperature began to occur. The linear shrinkage confirmed this trend—in the samples containing 15% of NS, linear shrinkage increased to 6.4%, but for samples with higher NS amounts, it is lower—4.9 and 4.2%. The compressive strength of the samples with higher SiO_2_/Na_2_O + K_2_O ratios (14.32 and 11.29) still increases, while the samples with SiO_2_/Na_2_O + K_2_O ratio of 9.1 remain almost unchanged, but in the samples with lower SiO_2_/Na_2_O + K_2_O ratios of 7.92 and 6.88, a significant decrease in compressive strength to 46.0 and 39.0 MPa was observed. Higher firing temperature continues to drive UPV growth in the samples, but the lowest UPV growth rate is observed in samples with the highest NS amounts. The apparent porosity and water absorption tend to increase; dilatometry curves confirm this. The decreases in these values indicate that the fluxing NS affected the pore size and structures of the samples (Figure 5). Porosity tests confirm this assumption.

### 3.3. Porosity

**Figure 8:**
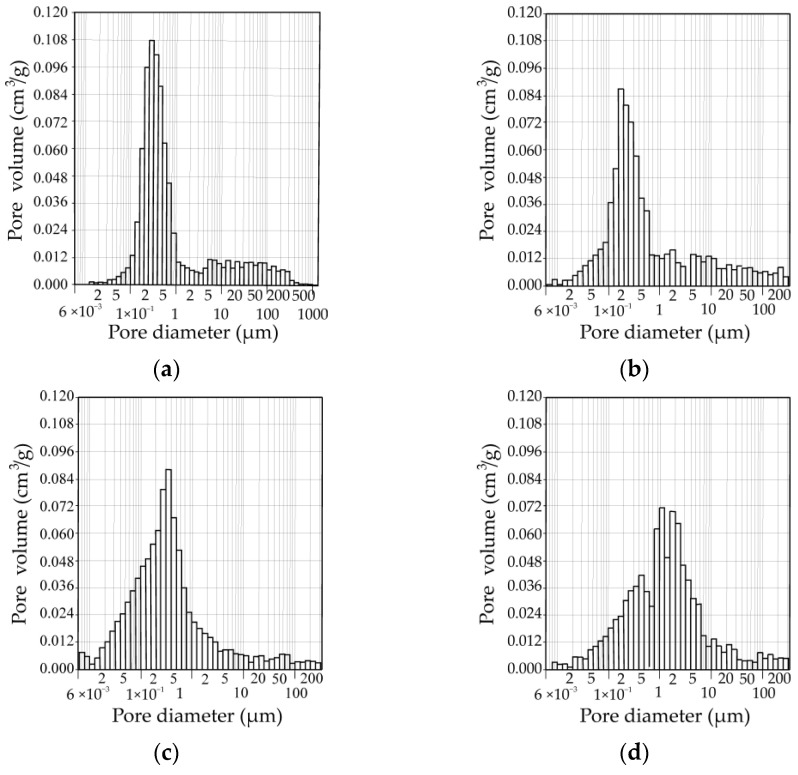
The pore size distribution (by volume) in the samples, depending on the amount of NS after firing at 1060 °C temperature: (**a**)—C_W_, (**b**)—C_W_N_5_, (**c**)—C_W_N_15_, (**d**)—C_W_N_25_.

**Figure 9:**
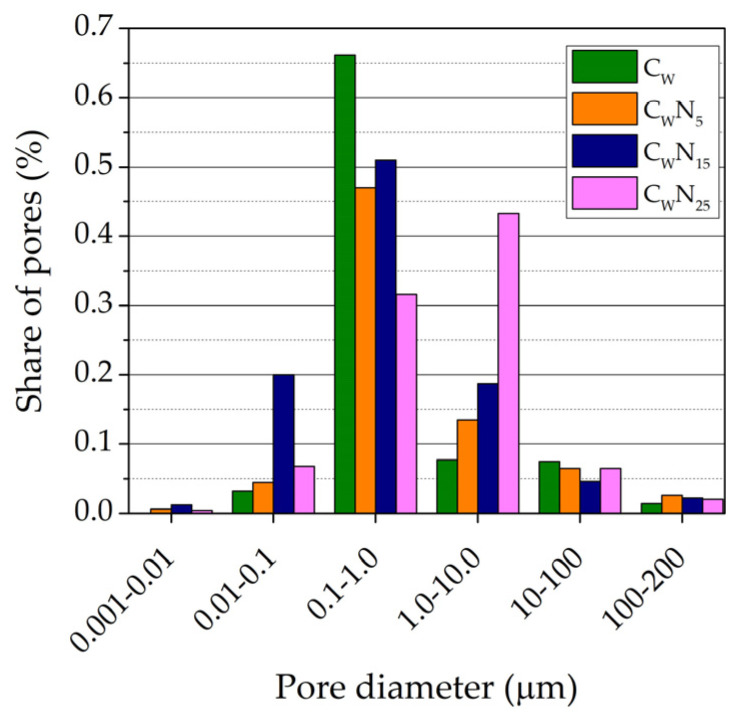
The pore size distribution (by percent) in the samples, depending on the amount of NS after firing at 1060 °C temperature.

The changes in the pore size distribution (by volume) in the samples depending on the amount of NS after firing at 1060 °C temperature can be seen in Figure 8. The summarised pore size distribution results are presented in Figure 9. According to the readings of the mercury porosimeter, in the C_W_ sample, pores with a diameter ranging from 0.01 to 0.10 µm occupied 0.032 cm^3^/g of a volume. The largest part of a volume—0.662 cm^3^/g was occupied by pores with a diameter ranging from 0.1 µm to 1 µm, while pores with a diameter ranging from 1.0 to 10 µm and 10.0 to 100 µm were occupied by 0.09 and 0.072 cm^3^/g volume. In the C_W_N_5_ sample, pores with a diameter ranging from 0.001 to 0.01 µm (0.006 cm^3^/g) appear, and pores with a diameter ranging from 0.01 to 0.10 µm occupy 30% higher volume (0.045 cm^3^/g). Similar to the C_W_ sample, the largest volume of 0.47 cm^3^/g is occupied by pores with a diameter ranging from 0.1 µm to 1.0 µm. However, contrary to the C_W_ sample, the volume of these pores is 1.88 times lower, and pores with a diameter ranging from 1.0 to 10 µm and 10.0 to 100 µm occupied higher 0.135 and 0.075 cm^3^/g volume. In the C_W_N_15_ sample, the volume from 0.001 to 0.01 µm in diameter pores increased by 2 times up to 0.012 cm^3^/g. The volume of pores, ranging from 0.01 to 0. 1 µm, increases more than 4.7 times—up to 0.209 cm^3^/g. The same tendencies were observed in porcelainised stoneware formulation with nepheline-syenite additive [37]. The author concludes that a significant fraction of pores under 0.1 µm is formed. Pores with a diameter ranging from 0.1 µm to 1 µm occupy the largest part of a volume—0.53 cm^3^/g; the volume of pores ranging from 1.0 µm to 10.0 µm tends to decrease. In the sample with the highest NS amount, the volume of the smallest pores (0.001–0.01 µm) tends to decrease by almost 3 times, and the pores with a diameter ranging from 0.1 µm to 1 µm decrease up to 1.7 times, compared to the C_W_N_15_ sample. Simultaneously, the volume of pores ranging from 1.0 to 10. 0 µm increased by almost 3.5 times and reached 0.445 cm^3^/g.

During the sintering process, melted particles of NS formed a vitreous phase that gradually decreased the open porosity of the ceramic samples and filled the voids, resulting from the MWMW fibre presence in the ceramic matrix (Figure 7a,b). Due to this interaction, the samples’ porosity and compressive strength were considerably enhanced. According to [68], due to the formation of the vitreous phase, the number of pores smaller than 0.1 µm decreased.

We can conclude that in composition with 15% of NS, due to an increase in active K^+^ and Na^+^ ions diffused into the ceramic matrix, the vitrification process promotes the formation of sufficiently large amounts of the smallest pore size from 0.001 to 0.01 µm and 0.01 to 0.10 µm. However, the apparent porosity values in the C_W_N_15_ sample are about 18% lower than in the reference sample. We can clearly see (Figure 8) that the pore size from 0.1 to 1.0 µm prevails in the reference sample, and the addition of NS adjusts the pore size and distribution in the samples. A lower amount of NS highly contributes to an increase in the part of the smallest pores with a diameter from 0.01 to 0.10 µm and 0.1 µm to 1.0 µm in the samples. The highest amount of NS creates larger pores from 1.0 µm to 10.0 µm.

### 3.4. Structural Parameters

The distribution of pores and, in particular, the formation of smaller pores has a positive effect on the frost resistance and durability of the sintered ceramic body [73]. This pore diameter distribution determines the lower density and UPV values of fired ceramic samples compared to the reference sample. These results confirmed the water absorption tests, showing that UPV decreased and water absorption increased. The increased amount of the smallest pores is particularly important since this amount determines the sintering degree of the ceramic samples, as water does not fill these pores, and thus affects the durability of the ceramic samples. Other authors [21,62] claim that it is not precise and hypothesize that pores larger than 3 µm in diameter benefit the frost resistance of ceramic samples. The literature mentions some indirect methods for the prediction and evaluation of the resistance of ceramic samples in freeze–thaw cycles. Well-known indirect methods for predicting the resistance of ceramic samples to freeze–thaw cycles are a calculation of structural parameters, damage indicator, and Maage factor [57].

**Figure 10:**
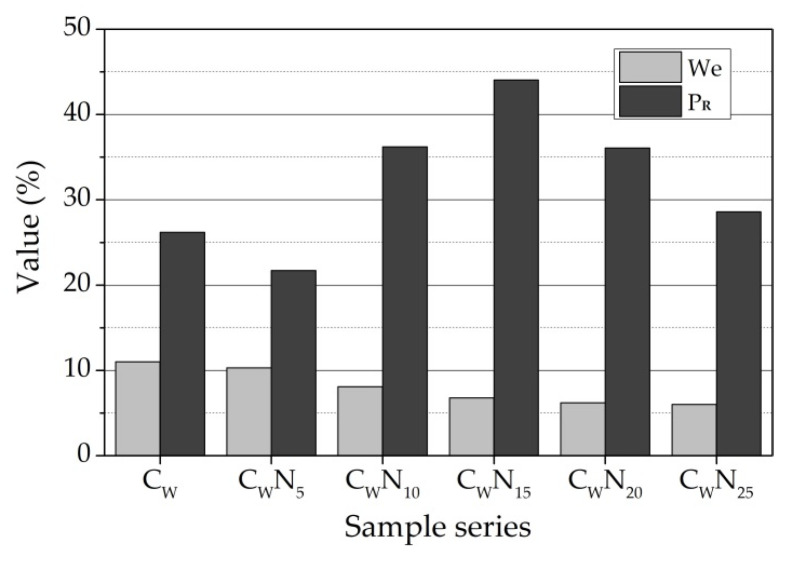
Structural parameters: effective porosity We and reserve of pore volume P_R_ of a ceramic body.

**Figure 11:**
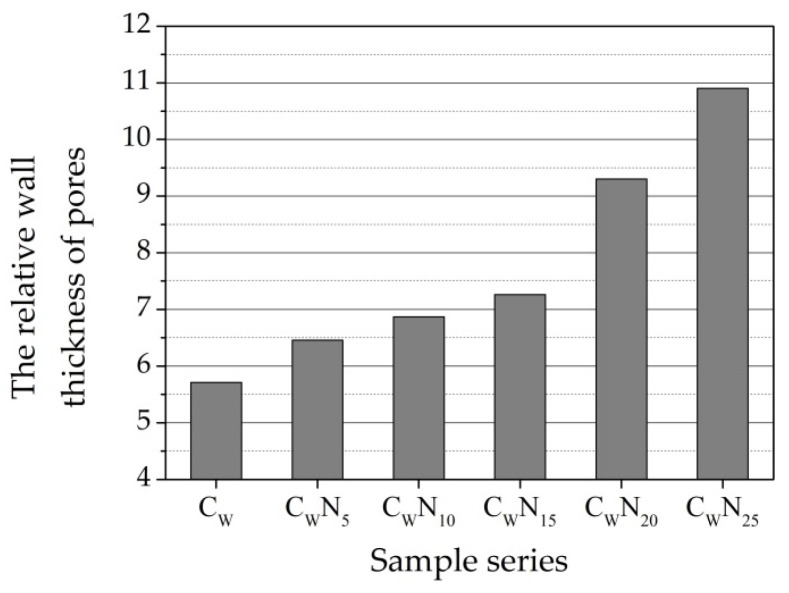
Structural parameters: relative wall thickness of pores D.

The porosity and structural parameters of a ceramic body are essential characteristics that determine the ceramic body’s durability, as well as mechanical and thermal conductivity properties [59,74,75]. Calculated structural parameters of ceramic samples of all compositions after firing at a temperature of 1060 °C are presented in (Figure 10 and Figure 11). The effective porosity of ceramic body defines the amount of effectively working pores and capillaries, and the reserve of pore volume represents part of the volume that penetrates water scarcely. Pores are filled gradually when samples are soaked in water and depend on the closed defective areas in the ceramic sample and the dimensions of pores and capillaries. The higher the reserve of pore volume, the higher the frost resistance of the ceramic bodies [60,75,76].

After burning at 1060 °C temperature, an increase in NS amount in the sample leads to a decrease in water absorption, apparent porosity, and the effective porosity We, while the reserve of pore volume P_R_, except for the C_W_N_5_ sample, sharply increases to 44.04 for C_W_N_15_ sample (Figure 10). Further increase in NS in C_W_N_20_ and C_W_N_25_ samples leads to a decrease in the reserve of pore volume in the sample. The acceleration of the sintering process can explain the intensification of agglomeration, the closing of open pores, and the decrease in open porosity. The lowest relative wall thickness D of pores is observed in the reference sample (Figure 11), and with the increase in NS amount in samples, the wall thickness of pores increases. The higher thickness of the wall of pores and capillaries predicts higher exploitation of frost resistance of ceramic samples.

According to the research in [57], frost resistance of ceramic samples depends upon pore geometry and, specifically, upon the proportion of larger than 3 µm in diameter pores, which do not fill up with water readily, to smaller pores, which absorb water susceptible to freezing under operating conditions. The pores larger than 3 µm in diameter positively influence the frost resistance of ceramic bodies. The Maage factor calculation involves the total volume of pores (PV) and the share of pores larger than 3 µm (P_3_—the percentage of the pores with diameters larger than 3 µm)). This provision was confirmed in the study carried out by [21]. Authors conclude that bricks without any sign of damage are characterised by the prevalence of pores with diameters in the range of 3–10 µm.

**Figure 12:**
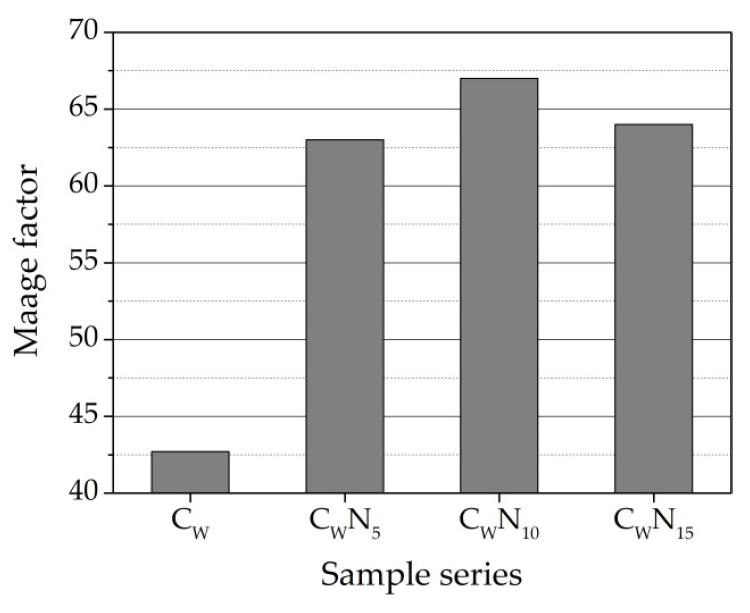
Calculated Maage factor for C_W_, C_W_N_5_, C_W_N_15_, and C_W_N_25_ samples.

The total volume of pores (P_V_) and P_3_ for the C_W_ sample is 0.747 cm^3^/g and 16.4%; for C_W_N_5_ sample is 0.718 cm^3^/g and 24.11%; for the C_W_N_15_ sample is 0.98 cm^3^/g and 26.4%; and for C_W_N_25_ sample is 0.92 cm^3^/g and 25.7%. Calculation of Maage factor F_C_ shows (Figure 12) that for the C_W_ sample, this value is 42.7; for C_W_N_5_, it is 63.0; for C_W_N_15_, it is 67.0; and for C_W_N_25_, it is 64.0. According to research in [24,62], ceramic samples with F_C_ values higher than 70 are durable. We can see that the C_W_N_15_ sample F_C_ value is very close to the specified limits, and values of C_W_N_5_ and C_W_N_25_ F_C_ samples are slightly lower. Only the C_W_ sample’s F_C_ value falls into a region where the frost resistance of the sample is uncertain. Nevertheless, the fact that the Fc of the C_W_N_15_ samples composition is higher than that of C_W_ and C_W_N_5_ samples only confirms the first structural parameters methodology results.

### 3.5. Frost Resistance Test

Samples of four compositions (C_W_, C_W_N_5_, C_W_N_15_, and C_W_N_25_) were obtained to test frost resistance after firing at a temperature of 1060 °C. Based on the observation that after firing at 1080 °C, swelling processes occur in the samples with higher NS content, and frost resistance tests were performed with samples fired at 1060 °C. Tests of the samples were carried out after 100, 200, and 300 cycles for a complete characterisation of changes in the structure of samples during cyclic freezing–thawing, visual inspection, UPV measurements, and compressive strength. The calculation of the damage indicator Ω after pointed cycles was performed.

**Figure 13:**
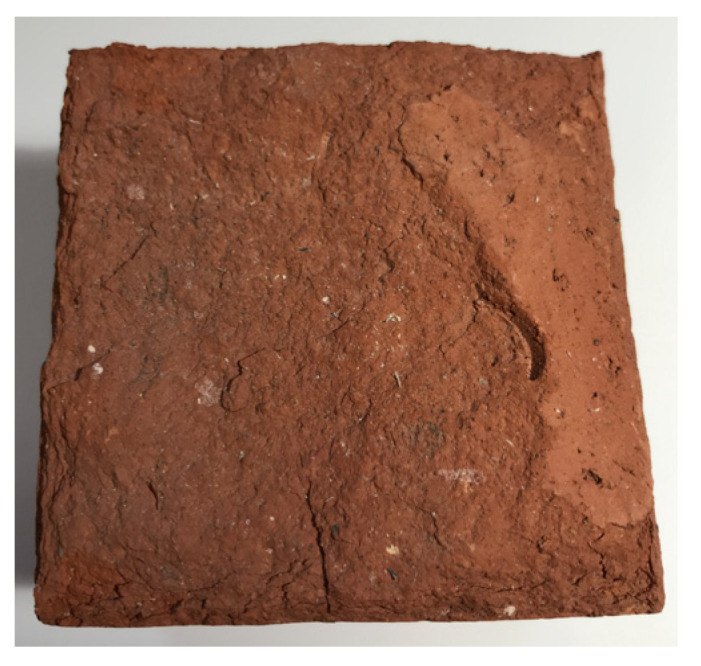
Visual inspection of the C_W_ samples after 300 freeze–thaw cycles.

A visual inspection of the samples after 100 and 200 freeze–thaw cycles revealed no destruction of samples of all compositions. After 300 freeze–thaw cycles, the surface of C_W_ samples was damaged (Figure 13), and no surface changes in the C_W_N_5_, C_W_N_15_, and C_W_N_25_ samples were observed.

**Figure 14:**
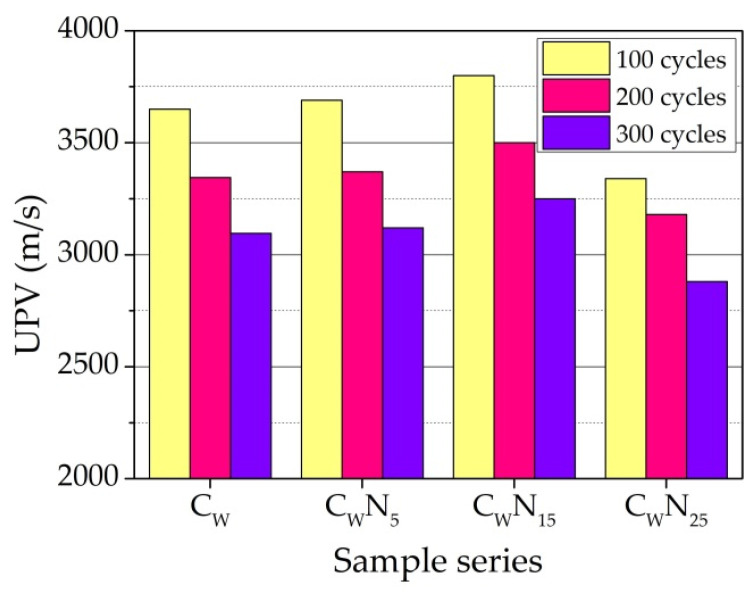
The UPV changes in the samples after burning and freeze–thaw cycles.

Compared to the UPV values after firing at a temperature of 1060 °C (Figure 5), it can be noted that the main decrease is in the UPV values, namely, the main destruction is observed after the first 100 freeze–thaw cycles of the samples (Figure 14). With the increase in the number of cycles, a further increase in UPV values is observed. However, the rate of increase in UPV values slows down. The influence of the amount of NS on changes in UPV during freezing–thawing can be evaluated by the percentage changes in UPV values compared with the values of UPV after firing at a temperature of 1060 °C. The decrease in the values of UPV after 100 freeze–thaw cycles in the samples of the composition C_W_ is 15.9%. In the samples of the compositions C_W_N_5_, C_W_N_15_, and C_W_N_25_, the UPV values are significantly lower—13.8, 8.7, and 9.3%. After 200 freeze–thaw cycles, the decrease in the values of UPV in comparison to UPV values after firing at a temperature of 1060 °C makes up 22.9, 21.3, 15.86, and 13.72%. After 300 cycles, the rate of destruction in the samples slows down even more; for the C_W_ samples, it is 28.7%, and for the C_W_N_5_—C_W_N_25_ samples, it is 27.1, 21.8, and 22.3%.

**Figure 15:**
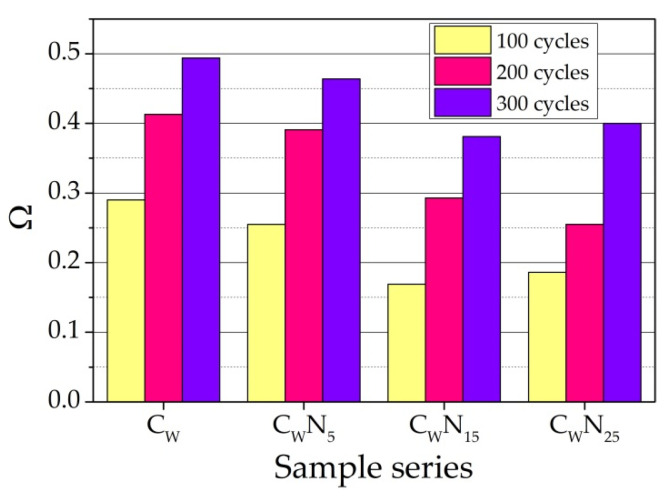
The damage indicator of the samples after burning and freeze–thaw cycles.

We hypothesize that an increase in the composition of the amount of NS to 5–25% due to the increase in active K^+^ and Na^+^ ions in the composition promotes a higher amount of small pore formation, which creates a more stable and better relaxing stress during freezing–thawing of the structure of the samples. An increase in the number of pores creates a more stable and better relaxing stress during the freeze–thaw structure of the samples. The calculation of damage indicator Ω confirms the assumptions and the UPV measurement results. As we can see, the damage is not severe after 100 freeze–thaw cycles, but C_W_ and C_W_N_5_ samples show the highest values. After 200 freeze–thaw cycles, the damage of C_W_ and C_W_N_5_ samples remains the highest and increases by 30%, while the damage of C_W_N_15_ and C_W_N_25_ samples increases by 42% and 27%. After 300 freeze–thaw cycles, the tendencies remain, but we can see that the damage indicator for all compositions increases by almost 2 times compared to values after 100 freeze–thaw cycles. Overall, it can be observed that the damage is less in the samples with higher NS amounts (Figure 15).

**Figure 16:**
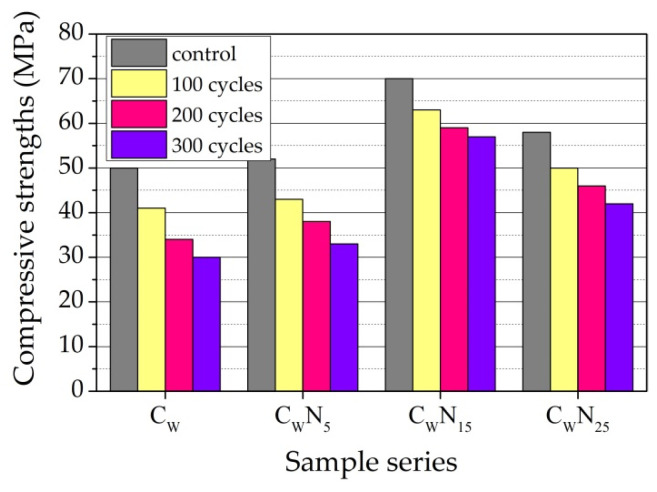
Compressive strength of the samples before and after freeze–thaw cycles.

Values of compressive strength of the ceramic samples before and after the exposure to freeze–thaw cycles are presented in Figure 16. Results show that residual compressive strength in the samples with NS is higher than in reference sample after 100–300 cycles. This is the consequence of the formed microstructure with a higher content of smaller pores and a lower content of larger (0.1–10 µm) pores. It is observed that the C_W_N_15_ sample best withstands exposure to freeze–thaw cycles.

**Figure 17:**
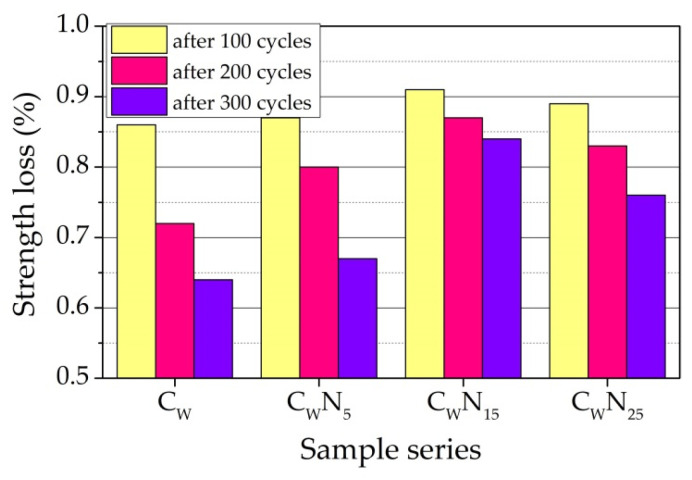
Loss of compressive strength of the samples after freeze–thaw cycles.

The calculation of loss of compressive strength, expressed as the ratio of compressive strength of the samples before and after freezing, was performed after 100, 200, and 300 cycles. Figure 17 shows that in the case of samples of composition C_W_ and C_W_N_5_, the loss of compressive strength after 100 cycles is very close—0.87 and 0.86, and in the case of samples of composition C_W_N_15_ and C_W_N_25_, the values are 0.91 and 0.89. After 200 cycles, the loss of compressive strength reaches 0.72 and 0.80 and 0.87 and 0,83, respectively. After 300 cycles, the loss of compressive strength reaches 0.64 and 0.67 for samples C_W_ and C_W_N_5_ and 0.84 and 0.76 for samples C_W_N_15_ and C_W_N_25_. It is noticed that an increase in NS amount in composition reduces the loss of compressive strength. The smallest loss of compressive strength is observed for sample C_W_N_15_.

This study confirms the positive effect of the addition of NS not only on the pore structure of the samples, but also on the residual strength of the samples. The study showed the possibility of using structural parameters and the ultrasound method to assess and predict ceramic samples’ frost resistance.

## 4. Conclusions

This study investigates the coupling effect of mechanically activated nepheline-syenite (NS) and mineral wool production waste on the physical–mechanical properties of a ceramic body. Based on the results of this research, it can be concluded that the optimum amount (10–20%) of mechanically activated fluxing additive NS positively affected the pore structure and physical, mechanical, and durability properties of ceramic samples prepared from low-melting illite clay and the mineral wool production waste when the ceramic samples were fired at 1000–1080 °C.

With an increase in NS in composition, the decrease due to an increased amount of alkaline compounds, such as Na_2_O and K_2_O with a greater tendency to vitrification, creates a glass phase during firing, which decreases the open porosity, modifies pore size distribution, and mainly enhances the compressive strength and frost resistance of the ceramic sample when the NS amount in the composition is 15%.

The lower amount of NS (up to 15%) highly contributes to an increase in the part of the smallest pore with a diameter from 0.01 to 0.10 µm and 0.1 µm to 1 µm in the samples. The highest amount of NS (25%) creates larger pores from 1.0 µm to 10.0 µm. Results showed that an increase in NS in composition up to 15% due to the modified pore structure after firing at 1060 °C enables the achievement of open porosity of 14.9–12.1%, density of 2120–2050 kg/m^3^, compressive strength of 50–70 MPa, linear shrinkage of 4.5–3.7%, and water absorption of 4.0–3.5%. Further increase in NS in composition up to 25% leads to a decrease in open porosity to 9.7–8.4%, ultrasonic pulse velocity of 3870–3680 m/s, density of 2090–2080 kg/m^3^, water absorption of 2.8–2.6%, linear shrinkage of 3.7–3.6%, compressive strength of 66–58 MPa, and density until 2090–2080 kg/m^3^. By increasing the NS amount from 5 to 15%, the reserve of pore volume increases from 26.2 to 44.1%, and the Maage factor increases from 42.7 to 67.0. Moreover, by increasing the NS amount to 20 and 25%, the reserve of pore volume decreases from 36.1 to 28.6%, and the Maage factor decreases to 64.

The frost resistance test shows that NS additive positively influences samples’ frost resistance. After 300 freeze–thaw cycles, surface damage is observed in the reference sample without NS additive. The UPV test results and damage indicator Ω results prove that the main destruction is observed after the first 100 freeze–thaw cycles of the samples. Furthermore, with the increasing number of cycles, the decrease in UPV values slows down. With the increase in NS amount in composition, the destruction rate in the samples decreases, and the value of the damage indicator decreases. It is observed that mainly the decrease in UPV and the highest value of damage indicator after freeze–thaw cycles are in the samples without NS additive.

It is established that an increase in NS amount in composition reduces the loss of compressive strength after freeze–thaw cycles. The smallest loss of compressive strength is observed for samples with 15% of NS.

This study shows the possibility of using structural parameters and the ultrasound method to assess and predict ceramic samples’ frost resistance.

## Figures and Tables

**Table 1:** Granulometric composition of components in %.

Components	Residue on Sieve (mm)%							
	2.0	1.0	0.5	0.25	0.125	0.08	0.045	<0.045
Clay	0.083	0.004	0.006	0.006	0.07	0.80	88.24	9.92
MWMW	-	-	0.29	3.92	19.62	39.55	26.84	9.79
NS	-	-	-	-	0.80	99.2	37.95	61.25

**Table 2:** The chemical composition of components, mass %.

Components	SiO_2_	Al_2_O_3_	Fe_2_O_3_	CaO	MgO	Na_2_O + K_2_O	Loss on Ignition
MWMW	42.13	18.30	5.81	16.15	13.94	1.73	1.40
Clay	46.88	17.93	5.31	10.36	4.37	2.67	14.50
NS	44.80	28.60	2.60	1.20	0.60	19.5	0.90

**Table 3:** The compositions of the forming masses.

Batch	Composition of Forming Masses, %		
	Clay	MWMW	NS
C_W_	80.0	20.0	-
C_W_N_5_	75.0	20.0	5.0
C_W_N_10_	70.0	20.0	10.0
C_W_N_15_	65.0	20.0	15.0
C_W_N_20_	60.0	20.0	20.0
C_W_N_25_	55.0	20.0	25.0

**Table 4:** Chemical composition of the forming masses and SiO_2_/Na_2_O + K_2_O and Al_2_O_3_/Na_2_O + K_2_O ratios in forming masses.

Batch	SiO_2_	Al_2_O_3_	Fe_2_O_3_	CaO	MgO	Na_2_O + K_2_O	SiO_2_/Na_2_O + K_2_O	Al_2_O_3/_Na_2_O + K_2_O
C_W_	51.38	20.06	5.94	12.96	7.03	2.62	19.61	7.66
C_W_N_5_	50.98	20.54	5.76	12.38	6.78	3.56	14.32	5.77
C_W_N_10_	50.59	21.02	5.58	11.81	6.5	4.48	11.29	4.69
C_W_N_15_	50.20	21.49	5.40	11.24	6.29	5.39	9.31	3.99
C_W_N_20_	49.81	21.96	5.22	10.68	6.05	6.29	7.92	3.49
C_W_N_25_	49.43	22.42	5.04	10.12	5.81	7.18	6.88	3.12

## Data Availability

Not applicable.
